# Case Report: Radiological characteristics and pathological correlation analysis of extranodal Rosai-Dorfman disease of liver

**DOI:** 10.3389/fonc.2025.1484820

**Published:** 2025-04-07

**Authors:** Xingxing Zheng, Hongzhe Tian, Wei Li, Meihong Yang, Chenwang Jin, Yuhui Pang

**Affiliations:** ^1^ Department of Medical Imaging, Baoji Central Hospital, Baoji, China; ^2^ Department of Medical Imaging, First Affiliated Hospital of Xi’an Jiaotong University, Xi’an, China; ^3^ Department of Pathology, Baoji Central Hospital, Baoji, China

**Keywords:** Rosai Dorfman disease, liver, imaging manifestations, pathological correlation analysis, enhanced scanning

## Abstract

**Background:**

Rosai Dorfman disease (RDD), also known as sinus histiocytosis with extensive lymph node involvement, is a rare histiocytosis with unknown etiology. Liver RDD is relatively rare and is often reported in individual cases. Exploring the imaging and pathological features of extrahepatic Rosai Dorfman disease (RDD) and conducting a comprehensive analysis of reported cases in domestic and foreign literature to enhance understanding of this rare disease.

**Methods:**

We collected data from a patient diagnosed with liver RDD in our hospital. In addition, we searched for liver RDD cases through PubMed and conducted a literature review.

**Results:**

Of the patient data used in this study, 1 patient’s data was obtained from our hospital records and 16 were retrieved from literature. There were 6 males and 11 females aged between 2-72 years, with an average age of 33.55 ± 19.38 years. Four patients presented with isolated liver nodules without the involvement of lymph nodes or extranodal organs, two patients presented with a case that involved extranodal organs but not lymph nodes, two patients presented with a case involving lymph nodes but not extranodal organs, eight patients presented with a case involving both lymph nodes and extranodal organs and lastly one patient presented with a case that did not involve either lymph nodes or extranodal organs. Four of the patients underwent enhanced scanning. The results revealed that 2 of them showed low enhancement, and the other 2 showed no significant enhancement. Only 1 case underwent PET-CT, and the results indicated a high uptake. Immunohistochemistry was performed on all 17 cases of liver RDD, and the results were positive for CD1a (-), CD68 (+), and S-100 (+).

**Conclusions:**

Liver RDD can manifest at any age. It presents with nonspecific imaging findings and a challenging preoperative diagnosis. Patients with extranodal hepatic RDD present with typical RDD characteristic immunohistochemistry features such as, high uptake on PET-CT, and no to mild enhancement on contrast-enhanced scans. For an early diagnosis, it is beneficial to fully comprehend these traits.

## Introduction

Rosai Dorfman disease (RDD), sometimes referred to as sinus histiocytosis with large lymphadenopathy (SHML), is a rare non-Langerhans cell histiocytosis that affects the extranodal areas and lymph nodes. Now often known as Rosai Dorfman disease or syndrome (RDD), it was first characterized by Azoury and Rreed. Later, Rosai and Ddorfman classified it as a benign course (1). Typical histological features of RDD include S-100 protein immunostaining positivity and CD1a negativity with tissue cell proliferation and epidermal hyperplasia. The most common site of RDD involvement is the lymph nodes, although extranodal involvement is occasionally noted. A case of extranodal intrahepatic RDD is one in which the liver is affected but no lymph nodes are involved. It is still quite uncommon, with most examples being documented as isolated incidents (2, 3). The most frequently observed extranodal locations are the skin, upper respiratory tract, and soft tissue. Additionally, reports of testes, thyroid gland, mammary glands, kidneys, and central nervous system involvement have been made (4, 5).

Therefore, this report aims to advance knowledge of RDD by comprehensively examining the imaging and clinical features of 17 patients who had been diagnosed with extranodal intrahepatic RDD.

## Patients and methods

### Patients

Relevant data of one patient with primary liver RDD confirmed by postoperative pathology in August 2023 were analyzed retrospectively and selected. Further 16 patients’ data diagnosed with liver RDD were collected after conducting a thorough literature review. The study was approved by the Ethics Committee of Shaanxi Province’s Baoji Central Hospital and written informed consent was waived due to the nature of the study.

### CT examination

The patient from our hospital had undergone an upper abdominal ultrasound examination before being transferred to the outpatient department. After hospitalization, plain and three-phase enhanced scans were performed on 256 row single source dual energy CT (GE, Revolution CT Xstream Edition). The parameters of the conventional sequence included instantaneous switching of tube voltage between 80 kVp and 140 kVp, tube current of 200-400 mA, layer thickness and spacing of 5 mm, reconstruction layer thickness of 1.25 mm, detector width of 80mm, tube rotation time of 0.5s, pitch of 0.992:1, and FOV of 35 cm x 35 cm. Iodohexanol (300 mgI/mL, dose of 1.2 mL/kg body mass) was used as the contrast agent for enhanced scanning and was injected at a rate of 3.0-3.5 mL/s through the anterior elbow vein. Following the injection of the contrast agent for 30 s, 60 s, and 120 s, arterial, venous, and delayed phase images were obtained and analyzed by two senior radiologists.

### Research design

The literature search using keywords “liver” and “Rosai Dorfman”, retrieved a total of 38 articles. After screening, a total of 16 cases of liver RDD were included. The confirmed liver RDD diagnosis data retrieved from our hospital was added, resulting in a total of 17 patients. The inclusion criteria for patients in this study included: (1) Patients diagnosed with liver RDD through pathological examination; (2) Case reports and literature related to liver RDD disease. The exclusion criteria included: (1) Case reports that did not include the full text of the literature, (2) duplicate reports, including the same case reported by the same author in different articles.

## Results

### Clinical manifestations

Of the 17 patients with liver RDD, 6 were male and 11 were female, ranging in age from 2 to 72 years, with an average age of (33.55 ± 19.38) years; the patient’s prognosis was usually good, with only 2 deaths from the disease (**Table 1**).

**Table 1:** Cases of Rosai–Rosai-Dorfman disease with liver involvement.

Reference	Age	Gender	Lymph Node Involvement	Other Site Involvement	Outcome
Present case	66	F	None	None	AW
Arabadzhieva et al (4)	58	F	Cystic lymph node, (Mascagni’s lymph node)	Gall-bladder	AW
Liu et al (20)	59	F	Left supraclavicular, mediastinal, supradiaphragmatic, hilar, and retroperitoneal regions	None	AWD
Roosta et al (3)	51	F	None	None	AWD
Gazia et al (2)	27	M	None	None	AWD
Yoshida1 et al (21)	74	F	Cervical, axillary, inguinal	Pelvis, heart, skin	AWD
Iwabuchi et al (22)	38 weeks	M	None	Splenomegaly	AWD
Shaikh et al (23)	59	F	None	Pancreas	LFU
Di et al (24)	65	F	None	None	AWD
Alruwaii et al (13)	68	M	–	–	–
Chen et al (25)	55	M	Superior phrenic, Hepatic hilar and retroperitoneal	bone marrow	LFU
Maheshwari et al (26)	3	F	Cervical, retroperitoneal, parahilar, paratracheal, perihepatic and para-aortic	None	AWD
Lauwers et al (27)	2.5	M	Supraclavicular, intra-abdominal	Bone, eye, thymus, heart, kidney, CNS, salivary land	DOD
Lauwers et al (27)	28	F	Cervical	CNS, breast	LFU
Lauwers et al (27)	0.7	F	Cervical, mediastinal, intra-abdominal, inguinal	Bone, bone marrow, orbit, trachea, skin, spleen, heart	DOD
Lauwers et al (27)	21	M	Cervical, hilar, axillary, inguinal	–	AWD
Lauwers et al (27)	7	F	Cervical, axillary, submandibular	Skin, omentum	AWD

M, male; F, female; LFU, lost at follow-up; AWD, alive with disease; DOD, dead of disease; AW, alive and well.

### Imaging findings

Results from the imaging analysis showed that 4 patients presented with a case of isolated liver nodules without the involvement of lymph nodes or extranodal organs, 2 patients presented with a case involving extranodal organs but not lymph nodes, 2 patients presented a case involving lymph nodes but not extranodal organs, 8 patients presented a case that involved lymph nodes and extranodal organs, and finally, 1 patient presented with a case without involvement of either lymph nodes and extranodal organs. Among them, 4 patients underwent enhanced scanning, of which 2 cases showed low enhancement and 2 cases showed no significant enhancement. Moreover, 2 patients underwent PET-CT and showed a high uptake. as illustrated in **Table 2**.

**Table 2:** The main imaging and pathologic features of extranodal RDD in the liver.

Reference	Imaging Features	Pathologic Features	Immunostains
Gazia et al (2)	Three focal hypodense hepatic lesions, with low marginal enhancement and blurred borders	Multinucleated histiocytic proliferation with emperipolesis	IHC staining detected a positive CD68 and S-100 protein with a negative CD1a
Iwabuchi et al. (22)	Decreased density of the right posterior region and the left lobe of the liver, and a narrowing of the left hepatic vein with peripheral thicking	Large multiform cells with round, oval, or grooved nucleus and eosinophilic cytoplasmwere observed. Marked hemosiderin depositions were shown in these atypical cells and emperipolesis was rarely observed	Positive for S-100 protein and CD68 but not CD1a
Roosta et al. (3)	Nonenhanced hypodense mass in the left lobe of the liver	Some dual or multinucleated occasionally represent a lymphophagocytosis	Histiocytes stained positively for CD68 marker and protein S-100 with immunohistochemistry(IHC) technique
Shaikh et al. (23)	A low-density mass in the left lobe of the liver with poorly defined boundaries and high PET-CT uptake	None	None
Di et al. (24)	Several hepatic nodules (ranging from 8 to 36 mm)	The hepatic nodule was composed of large cells with eosinophilic cytoplasms and large vesicular nuclei, characterized by diffuse lymphocytophagocytosis	Strong immunoreactivity for S-100 protein, negative for CD45, CD68, CD1a, CD30
Present case	Moderately uneven enhanced nodules in the left lobe of the liver	A large number of lymphoid tissue hyperplasia with lymphoid follicle formation	Strong immunoreactivity for S-100 protein, CD68, CD20, CD163, negative for CD1a

### Organizational pathological manifestations

A significant quantity of lymphoid tissue proliferation was detected in all 17 individuals, as well as the development of lymphoid follicles. A substantial amount of plasma cells and abundant light-stained cytoplasm were found between the follicles, with huge oval-shaped cells clustered together. Lymphocytes were detected in the cytoplasm in affected cells, and neutrophils, lymphocytes, and plasma cells with intact phagocytic morphology were locally observed. The results from immunohistochemistry showed patients presented with classic immunophenotypical results which were the expression of CD68 (+), and S-100 (+) but not CD1a (-) (**Table 2**).

## Case report

**Figure 1:**
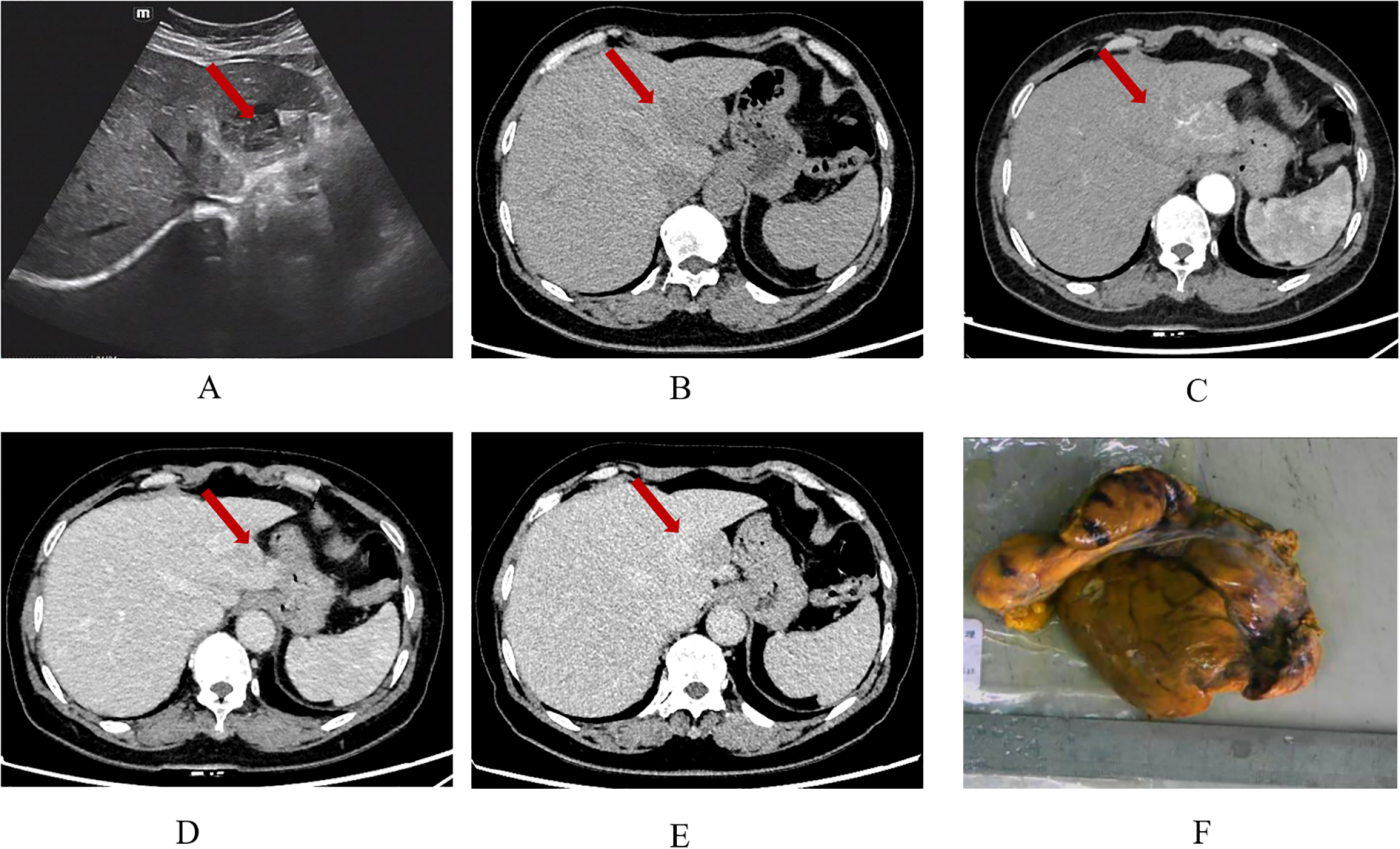
Ultrasound shows a hypoechoic nodule in the left outer lobe of the liver, with clear boundaries and uneven internal echoes **(A)**; CT plain scan **(B)** shows a low-density shadow of about 3.0 x 2.9 cm in size in the left lobe of the liver, with smooth edges and clear boundaries. The plain scan density is uniform, with a CT value of about 49 HU. In the arterial phase **(C)** of the enhanced scan, the lesion shows moderate and uneven enhancement, with a CT value of about 77 HU. The accessory hepatic artery originating from the left gastric artery can be seen to participate in blood supply. In the portal phase **(D)**, the lesion is further enhanced, with a CT value of about 87 HU. In the delayed phase **(E)**, the enhancement slightly decreases, with a CT value of about 75 HU. Gross examination of the tumor specimen **(F)**.

**Figure 2:**
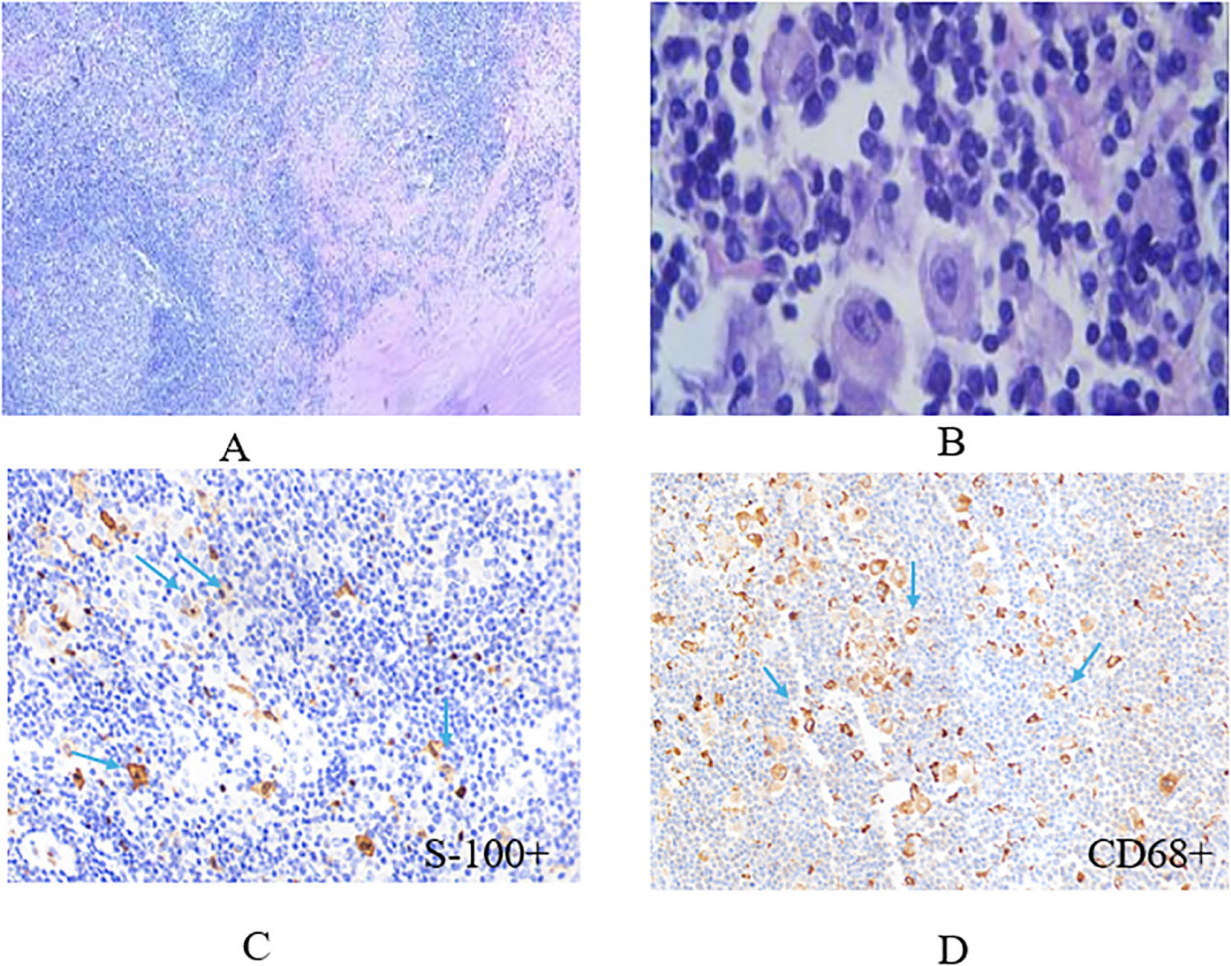
The representative image of histiocytes with eosinophilic nucleolus stained by hematoxylin and eosin (HE) method. Some dual or multinucleated occasionally represent a lymphophagocytosis (emperipolesis, left image: x10, right image: x40) **(A, B)**, histiocytes stained positive for S-100 marker and CD68 with immunohistochemistry (IHC) technique (x40) **(C, D)**.

The patient diagnosed with isolated RDD in the left lobe of the liver was a 66-year-old woman. Results from the Ultrasound showed a hypoechoic mass in the left lobe of the liver with clear boundaries. Color Doppler flow imaging (CDFI) revealed a small amount of blood flow signal around the mass (**Figure 1A**), and CT results showed an exogenous nodule of approximately 3.0 cm x 2.9 cm in size in the left outer lobe of the liver, with smooth edges, clear boundaries, and uniform density. Furthermore, the enhanced scan showed mild enhancement in the arterial phase, sustained weak enhancement in the portal phase, and slightly reduced enhancement in the delayed phase. There was no sign of cystic necrosis or external invasion within the lesion, and the adjacent perihepatic space was clear (**Figures 1B–E**). Significant lymphoid tissue proliferation was observed under the microscope, along with the development of different-sized lymphoid follicles. In the interstitial region of the follicles, a large number of plasma cells and an abundance of rich, pale cytoplasm were visible, and huge, round-nucleated cells were clustered together. **Figure 2** shows the presence of lymphatic cells in the cytosol. The affected cells expressed CD68 (+) and S-100 (+), but not CD1a (-) as illustrated by immunohistochemistry analysis.

## Discussion

Rosai Dorfman disease was defined as a lymphohematopoietic system malignancy by the WHO in 2006. In the 2016 updated classification of tissue cells and tumors, RDD is categorized as the R group, which includes familial, classical (lymph node type), extranodal, tumor-related, and autoimmune disease-related types (6–8). Numerous theories regarding the pathophysiology of RDD have been established, but its etiology remains unclear. Various etiologies have been speculated to cause RDD and these include immunodeficiency, infectious factors, autoimmune diseases, instability in the cellular microenvironment, and genetic mutations (9–11).

RDD can occur at any age but is more common in children and adolescents. This disease mainly affects African American males with a male-to-female ratio of 3:1 (12). From our findings, the 66-year-old woman presented with a classic type of RDD, belonging to the extranodal type. The lymph nodes were affected bilaterally, they were large painless cervical lymph node disease with or without intermittent fever. The patient experienced night sweats and weight loss. In some cases, mediastinal, axillary, and inguinal lymph nodes may also be affected, but retroperitoneal lymph nodes are rarely affected. Externally, the skin, soft tissue, upper respiratory tract, bones, orbit, nasopharynx, salivary glands, central nervous system, testes, and endocrine glands were the most severely affected areas (4, 13).

With non-specific imaging features, liver RDD is frequently misinterpreted as granulomatous lesions or as other tumor-related lesions. It may show up as isolated or many, single or multiple diffuse liver lesions. Six of the 17 cases in this group had masses or nodules in the liver that were slightly low-density; the isolated liver nodules were all located in the left lobe of the liver. Three of the cases had liver lesions that were isolated, two cases presented with multiple liver lesions (3 or more), and one case had diffuse liver lesions (reduced T2WI signal and narrowing of the internal bile duct). Among them, 4 cases underwent enhanced scanning, of which 2 cases showed low enhancement, and the other 2 cases showed no significant enhancement.

It is necessary to differentiate RDD from the following illnesses when it manifests as single nodules in the liver; (1) Mass or nodular lymphoma: As a common hypovascular tumor, liver lymphoma often exhibits gradually mild to moderate enhancement on dynamic contrast-enhanced scans, with a rather uniform enhancement. Since lymphoma develops in the liver interstitium, certain cancers may exhibit intrinsic liver blood vessels on contrast-enhanced imaging, which frequently results in the so-called “vascular floating syndrome (14, 15).” The surrounding blood vessels around the lesion are primarily characterized by narrowing, compression, and deformation with visible continuous vascular shadows (a classic manifestation of lymphoma. (2) Intrahepatic cholangiocarcinoma: This cancer manifests as low-density on plain scan. It causes adjacent liver capsule retraction, surrounding bile duct dilation, arterial phase peripheral ring enhancement, and gradual centripetal enhancement at the lesion center. The DWI shows target signs, indicating inconsistency between peripheral and internal information. Gadolinium acid cloud, with a high signal in the center and low signal in the periphery of liver and gallbladder lesions, represent a large number of live tumor cells in the surrounding area and varying degrees of fibrosis in the center (16). (3) Atypical hepatocellular carcinoma: When hepatocellular carcinoma does not have fast in and out typical enhancement features, it is difficult to diagnose. In China, liver cancer often occurs based on the development of hepatitis and cirrhosis. The combined effect of a medical history and AFP examination, can to successfully differentiate this disease (17).

To diagnose RDD, histopathological/immunohistochemical investigations are regarded as the gold standard. The findings from these analyses demonstrate that the afflicted liver’s overall mass is solid, with gray, white-gray cross-sections and a relatively hard texture. Certain areas of the liver may have a capsule. Low magnification reveals an uneven distribution of light and dark in the lesion area, while high magnification reveals tissue and cell proliferation in the bright area, with cells showing up as oval or polygonal in form, with plenty of cytoplasm, light staining, or eosinophilic red. A significant amount of atypia is present in the cells, and distinct binucleated cells and nucleoli are apparent. significant tissue cells also show different-sized vacuoles (18). In all 17 cases of this article, a significant amount of lymphoid tissue proliferation along with the development of lymphoid follicles was observed. Between the follicles, there were numerous plasma cells, a significant amount of cytoplasm, and large, oval-shaped cells with nuclei clustered together. Neutrophils, lymphocytes, and plasma cells with full phagocytic morphology were locally observed, and lymphocytes were discovered in the cytoplasm. For the diagnosis of RDD, immunohistochemical testing is crucial. While CD1a, CD207, and CD34 are not expressed by tissue cells, they frequently express S-100, CD68, and other proteins. S-100 protein (+) plays a significant role in the development of this disease (19). In 17 RDD tissues examined in this investigation, S-100, CD68, and CD163 were all positive whereas CD1a was negative.

In conclusion, extramedullary RDD of the liver is rare and only a few reports have been documented in the literature. Most of these reports have only provided a single aspect of either pathology or imaging. This study combines imaging and pathology to introduce the case and compares it with previous literature reports to comprehensively evaluate the disease and enhance our understanding of it.

## Data Availability

The original contributions presented in the study are included in the article/supplementary material. Further inquiries can be directed to the corresponding authors.
